# Immunoprophylaxis against respiratory syncytial virus with palvizumab: what is new?

**DOI:** 10.1590/0103-05822014322

**Published:** 2014-06

**Authors:** Marco Aurélio P. Sáfadi

**Affiliations:** 1 FCMSCSP, São Paulo, SP, Brasil

Infection by the respiratory syncytial virus (RSV) is recognized as the most important cause of respiratory tract infection in infants and young children in the world, being the main responsible for hospitalizations during winter in the first year of life^(^ 1 ^)^. However, despite the importance of RSV as a cause of morbidity and mortality in children, there are still no effective vaccines or antiviral drugs to prevent and treat this infection. The disastrous experience with an inactivated vaccine candidate made with whole virus, developed in the sixties, still unrests the medical community in the search for a safe and effective vaccine against RSV infection^(^ 2 ^)^. The possibility of preventing the infection by RSV gained even more importance in the last 10 years, due to the accumulation of evidence showing that hospitalized infants with lower respiratory tract infection (especially those caused by RSV and rhinovirus) are at greater risk of developing recurrent episodes of wheezing and asthma, when compared to children that did not present severe bronchiolitis^(^ 3 ^-^ 5 ^)^.

Passive immunization with palivizumab, a humanized monoclonal antibody, is currently the main tool available for the prophylaxis of RSV infection. Its use is indicated for specific risk groups: preterm infants and children under 2 years old with congenital heart disease and hemodynamic repercussion or with chronic pulmonary disease, who needed treatment in the 6 months prior to the season of RSV^(^ 6 ^)^. However, the impact of this intervention in the burden of the disease attributable to RSV is minimal, once most hospitalizations due to the virus occur in healthy infants, who are not included in prevention programs.

The current edition of the Journal includes an article by Monteiro et al^(^ 7 ^)^, which presents results of a study including a cohort of 198 infants who received palivizumab, according to the recommendations in the state of São Paulo, Southeast Brazil. Infants were followed prospectively during a year, to assess the impact of palivizumab on the viral etiology of acute infections in the respiratory tract and rates of hospitalization and death. It is remarkable, among the various important findings, the rate of only 0.7% of hospitalization by RSV in infants undergoing immunoprophylaxis (only one hospitalization by RSV among the 198 followed infants). One of the limitations of the study, pointed by the authors, was the lack of a control group, for ethical reasons, which prevented the assessment of the effectiveness of the intervention with immunoprofilaxys in the studied group.

There is today a growing debate regarding the scope of indications for the use of palivizumab, motivated not only by the high cost of the product, but also by recent evidence in the literature. The licensing of palivizumab, in the late 1990s, was based on the results of a randomized, placebo-controlled study with 1,500 preterm infants, which showed that immunoprofilaxys reduced in approximately 55% the rates of hospitalization by RSV (10.6% in the Placebo Group against 4.8% among high-risk infants who received palivizumab)^(^ 8 ^)^. However, more recent data^(^ 9 ^-^ 11 ^)^ suggest that the RSV hospitalization rates among preterm infants belonging to risk groups present a trend towards reduction in relation to the rates found in the studies conducted in the 1990s, in line with the results by Monteiro et al in Brazil.

The confirmation of lower rates of de hospitalization by RSV in preterm infants currently brings a new challenge for the analysis of the cost-effectiveness demonstration of this intervention, requiring an even higher number on infants covered with immunoprofilaxys to enable the prevention of one RSV hospitalization. On the other hand, there is increasing evidence suggesting that the use of palivizumab in children reduces the incidence of future episodes and days of wheezing^(^ 12 ^,^ 13 ^)^, anticipating the possibility of additional benefits, not incorporated into cost-effectiveness and that, if confirmed in other populations, may change the scenario of the prevention of the infection by RSV.

Finally, we emphasize that there is no evidence so far in the literature to support the extending use of palivizumab to other groups, such as patients presenting cystic fibrosis, those immunocompromised, and patients with Down syndrome, nor to support its use to control outbreaks in Neonatal Intensive Care Units. Therefore, palivizumab does not have indication for routine use in these situations^(^ 14 ^)^.

## References

[1] Hall CB, Weinberg GA, Iwane MK, Blumkin A, Edwards KM, Staat M (2009). The burden of respiratory syncytial virus infection in
young children. N Engl J Med.

[2] Chin J, Magoffin RL, Shearer LA, Schieble JH, Lennette EH (1969). Field evaluation of a respiratory syncytial virus
vaccine and a trivalent parainfluenza virus vaccine in a pediatric
population. Am J Epidemiol.

[3] Stein RT, Sherrill D, Morgan WJ, Holberg CJ, Halonen M, Taussig LM (1999). Respiratory syncytial virus in early life and risk of
wheeze and allergy by age 13 years. Lancet.

[4] Sigurs N, Gustafsson PM, Bjarnason R, Lundberg F, Schmidt S, Sigurbergsson F (2005). Severe respiratory syncytial virus bronchiolitis in
infancy and asthma and allergy at age 13. Am J Respir Crit Care Med.

[5] Caliskan M, Bochkov YA, Kreiner-Møller E, Bonnelykke K, Stein M, Du G (2013). Rhinovirus wheezing illness and genetic risk of
childhood-onset asthma. N Engl J Med.

[6] Pickering LK, Baker CJ, Kimberlin DW, Long SS, AAP Committee on Infectious Diseases (2012). Respiratory syncytial virus. Red Book: Report of the Committee on Infectious
Diseases.

[7] Monteiro AI, Bellei NC, Sousa AR, Santos AM, Weckx LY (2014). Infecções respiratórias em crianças menores de dois anos
de idade submetidas à profilaxia com palivizumabe. Rev Paul Pediatr.

[8] (1998). Palivizumab, a humanized respiratory syncytial virus
monoclonal antibody, reduces hospitalization from respiratory syncytial virus
infection in high-risk infants. The IMpact-RSV Study Group. Pediatrics.

[9] Zhou H, Thompson W, Viboud C, Ringholz C, Cheng PY, Steiner C (2012). Hospitalizations associated with influenza and
respiratory syncytial virus in the United States, 1993-2008. Clin Infect Dis.

[10] Paes B, Mitchell I, Li A, Harimoto T, Lanctôt KL (2013). Respiratory-related hospitalizations following
prophylaxis in the Canadian registry for palivizumab (2005-2012) compared to other
international registries. Clin Dev Immunol.

[11] Hasegawa K, Tsugawa Y, Brown DF, Mansbach JM, Camargo CA (2013). Trends in bronchiolitis hospitalizations in the United
States, 2000-2009. Pediatrics.

[12] Yoshihara S, Kusuda S, Mochizuki H, Okada K, Nishima S, Simões EA, C-CREW Investigators (2013). Effect of palivizumab prophylaxis on subsequent
recurrent wheezing in preterm infants. Pediatrics.

[13] Blanken MO, Robers MM, Molenaar JM, Winkler-Seinstra P, Meijer A, Kimpen JL (2013). Respiratory syncytial virus and recurrent wheeze in
healthy preterm infants. N Engl J Med.

[14] Meissner HC, Kimberlin DW (2013). RSV immunoprophylaxis: does the benefit justify the
cost?. Pediatrics.

