# Supplementary material for: Immunoprophylaxis against respiratory syncytial virus with palvizumab: what is new?
Source: Rev Paul Pediatr. 2014 Jun;32(2):150–1. doi: 10.1590/0103-05822014322 (PMC4183015; doi:10.1590/0103-05822014322)
Supplement: Translation

# Translation: Imunoprofilaxia do vírus sincicial respiratório com palivizumabe: o que há de novo?

**Authors:** Marco Aurélio P. Sáfadi

**Affiliations:** 1 FCMSCSP, São Paulo, SP, Brasil

A infecção pelo vírus sincicial respiratório (VSR) é reconhecida como a mais importante causa de infecção do trato respiratório em lactentes e crianças pequenas no mundo, sendo a principal responsável por hospitalizações durante o inverno no primeiro ano de vida^(^ ^1^ ^)^. Entretanto, a despeito da importância do VSR como causa de morbidade e mortalidade em crianças, ainda não dispomos de uma vacina ou de drogas antivirais eficazes para prevenir e tratar essa infecção. A desastrosa experiência com uma vacina candidata inativada feita com o vírus inteiro, desenvolvida nos anos 1960, até hoje inquieta a comunidade médica na busca por uma vacina segura e eficaz contra a infecção pelo VSR^(^ ^2^ ^)^. A possibilidade de poder prevenir a infecção pelo VSR ganhou ainda mais importância nos últimos dez anos, a partir do acúmulo de evidências demonstrando que lactentes hospitalizados com infecção do trato respiratório inferior (especialmente as causadas pelo VSR e pelo rinovírus) têm maior risco de desenvolver episódios recorrentes de sibilância e asma, quando comparados a crianças que não tiveram quadros severos de bronquiolite^(^ ^3^ ^-^ ^5^ ^)^.

A imunização passiva com o palivizumabe, um anticorpo monoclonal humanizado, é hoje a principal ferramenta disponível para a profilaxia da infecção pelo VSR. Sua utilização é indicada para grupos de risco específicos: lactentes prematuros e crianças menores de dois anos portadoras de cardiopatia congênita com repercussão hemodinâmica ou com doença pulmonar crônica, que necessitaram de tratamento nos seis meses anteriores ao período da sazonalidade do VSR^(^ ^6^ ^)^. Entretanto, o impacto dessa intervenção na carga da doença atribuída ao VSR é mínimo, uma vez que a maioria das hospitalizações pelo vírus ocorre em lactentes saudáveis, não incluídos nos programas de prevenção.

Nesta edição da Revista, Monteiro *et al**^(^* ^7^ ^)^ apresentam os resultados de um estudo que incluiu uma coorte de 198 lactentes que receberam palivizumabe, segundo as recomendações vigentes no Estado de São Paulo. Os lactentes foram acompanhados prospectivamente, durante um ano, para avaliar o impacto do palivizumabe na etiologia viral das infecções agudas do trato respiratório e nas taxas de internação e óbito em crianças submetidas a essa imunoprofilaxia.

Merece destaque, entre os vários importantes achados, a taxa de apenas 0,7% de hospitalização por VSR nos lactentes submetidos à imunoprofilaxia (uma única internação por VSR entre os 198 lactentes seguidos). Uma das limitações do estudo, apontada pelos autores, foi a ausência da inclusão, por razões éticas, de um grupo controle, o que impossibilitou avaliar a eficácia da intervenção com a imunoprofilaxia no grupo estudado.

Existe hoje um debate crescente quanto à abrangência das indicações do uso do palivizumabe, motivado não só pelo elevado custo do produto, mas também por recentes evidências surgidas na literatura. O licenciamento do palivizumabe, no final dos anos 1990, baseou-se no resultado de um estudo randomizado, controlado com placebo, com 1.500 lactentes prematuros, que mostrou que a imunoprofilaxia reduziu em aproximadamente 55% as taxas de hospitalização por VSR (10,6% no Grupo Placebo contra 4,8% entre os lactentes de alto risco que receberam palivizumabe)^(^ ^8^ ^)^. Entretanto, dados mais recentes^(^ ^9^ ^-^ ^11^ ^)^ sugerem que as taxas de hospitalização por VSR em lactentes prematuros pertencentes aos grupos de risco apresentam tendência de redução em relação às taxas encontradas nos estudos realizados nos anos 1990, em consonância com os resultados de Monteiro *et al* no Brasil.

A confirmação de menores taxas de hospitalização por VSR nos lactentes prematuros atualmente traz, portanto, um novo desafio para as análises de demonstração de custo-efetividade dessa intervenção, fazendo com que seja necessário um número ainda maior de lactentes contemplados com a imunoprofilaxia para possibilitar a prevenção de uma internação por VSR. Por outro lado, acumulam-se evidências sugerindo que o uso do palivizumabe em crianças reduz a incidência futura de episódios e dias de sibilância^(^ ^12^ ^,^ ^13^ ^)^, antecipando a possibilidade de benefícios adicionais, não incorporados em análises de custo-efetividade e que, se confirmados em outras populações, podem mudar o cenário da prevenção da infecção pelo VSR.

Finalmente, destacamos que não há, até o momento, evidências na literatura que suportem a ampliação do uso do palivizumabe a outros grupos, como pacientes com fibrose cística, imunocomprometidos e portadores de síndrome de Down, assim como a sua utilização no controle de surtos em Unidades de Terapia Intensiva Neonatal, não existindo, portanto, indicação rotineira para seu uso nessas situações^(^ ^14^ ^)^.
